# Supplementary material for: Comprehensive Annotation and Functional Exploration of MicroRNAs in Lettuce
Source: Front Plant Sci. 2021 Dec 24;12:781836. doi: 10.3389/fpls.2021.781836 (PMC8739914; doi:10.3389/fpls.2021.781836)
Supplement: Supplementary file 2 [file Data_Sheet_8.ZIP › GSM2230749.plot/Lsa-miRN55_Lsat_1_v5_gn_5_130440.1_100_TPlot.pdf]

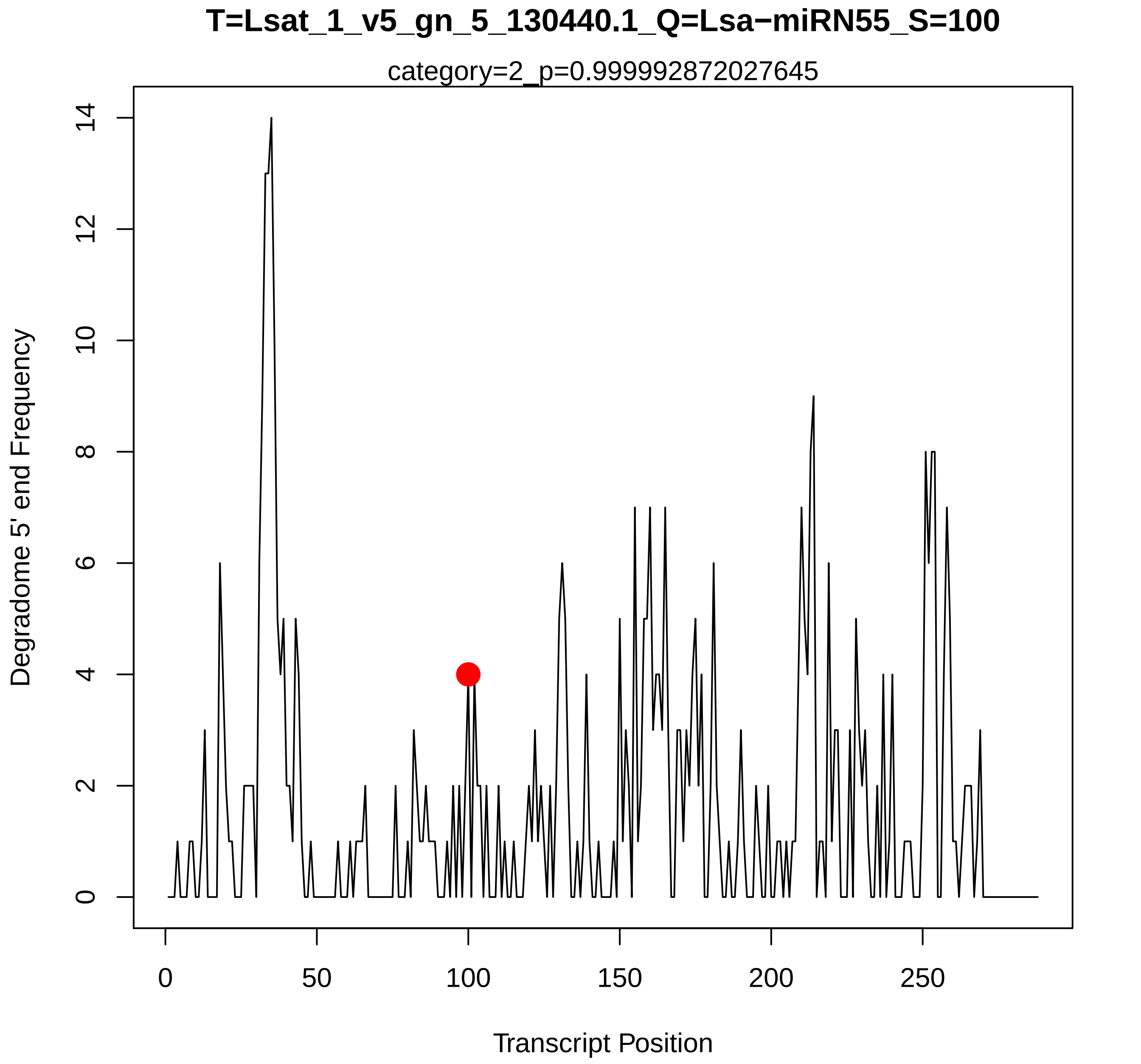

T=Lsat_1_v5_gn_5_130440.1_Q=Lsa−miRN55_S=100
category=2_p=0.999992872027645
Degradome 5' end Frequency
0
2
4
6
8
10
12
14
0
50
100
150
200
250
Transcript Position
